# 50th Anniversary of the First Surgeon General’s Report on Smoking and Health

**Published:** 2014-01-17

On January 17, 2014, 50 years after the first Surgeon General’s report on smoking and health, the Surgeon General will release *The Health Consequences of Smoking—50 Years of Progress* (1). The report will be released at a White House press conference at 9:30 a.m. Eastern on January 17, with a live webcast available at http://whitehouse.gov/live.

The report will provide a retrospective of the past 50 years of tobacco use prevention and control efforts and discuss the current status of the tobacco use epidemic, including newly documented health consequences of smoking and exposure to secondhand smoke. It will also include a discussion of potential “endgame” strategies to eliminate the health and economic burden of tobacco use in the United States.

Evidence-based tobacco control efforts have averted nearly 8 million deaths since 1964 but remain underutilized (2). High-impact media campaigns, excise taxes, access to cessation medication and counseling, smoke-free laws, comprehensive tobacco control programs, and product regulation are critical to ending the tobacco use epidemic.
